# Supplementary material for: Dopamine regulates decision thresholds in human reinforcement learning in males
Source: Nat Commun. 2023 Sep 4;14:5369. doi: 10.1038/s41467-023-41130-y (PMC10477234; doi:10.1038/s41467-023-41130-y)
Supplement: Supplementary file 1 — Supplementary Information [file 41467_2023_41130_MOESM1_ESM.pdf]

Supplemtary information for

Dopamine regulates decision thresholds in human reinforcement learning in males.

Karima Chakroun[1], Antonius Wiehler[2], Ben Wagner[3], David Mathar[4], Florian Ganzer[5], Thilo vanEimeren[6], Tobias Sommer[1] & Jan Peters[1,4]

[1]Institute for Systems Neuroscience, University Medical Center Hamburg-Eppendorf, Hamburg, Germany

[2]Motivation, Brain and Behavior Lab, Paris Brain Institute (ICM), Pitié-Salpêtrière Hospital, Paris, France

[3]Chair of Cognitive Computational Neuroscience, Technical University Dresden, Dresden, Germany.

[4]Department of Psychology, Biological Psychology, University of Cologne, Cologne, Germany

[5]Integrated Psychiatry Winterthur, Winterthur, Switzerland.

[6]Multimodal Neuroimaging Group, Department of Nuclear Medicine, University Medical Center Cologne, Cologne, Germany.

Corresponding author:

Jan Peters jan.peters@uni-koeln.de

Table S1. Overview of priors for group-level means.

**Parameter**		**Mean (placebo condition)**	**Mean (drug effects)**
$\boldsymbol{\alpha}$	*Boundary separation*	*U(.01, 5)*	$\mathcal{N}(0, 2)$
$\boldsymbol{\tau}$	*Non-decision time*	*U(.1, 2)*	$\mathcal{N}(0, 2)$
$\boldsymbol{v_{coeff}}$	*Drift rate coeff*	*U(-100, 100)*	$\mathcal{N}(0, 2)$
$\boldsymbol{\eta_{+}, \eta_{-}}$	*Learning rates*	*U(-5,5)*	$\mathcal{N}(0, 2)$

Table S2. Overview of priors for group-level standard deviations.

**Parameter**		**SD (placebo condition)**	**SD (drug effects)**
$\boldsymbol{\alpha}$	*Boundary separation*	*U(1e-4, 2)*	*U(1e-4, 2)*
$\boldsymbol{\tau}$	*Non-decision time*	*U(1e-4, 2)*	*U(1e-4, 2)*
$\boldsymbol{v_{coeff}}$	*Drift rate coeff*	*U(1e-4, 10)*	*U(1e-4, 10)*
$\boldsymbol{\eta_{+}, \eta_{-}}$	*Learning rates*	*U(1e-4, 4)*	*U(1e-4, 4)*

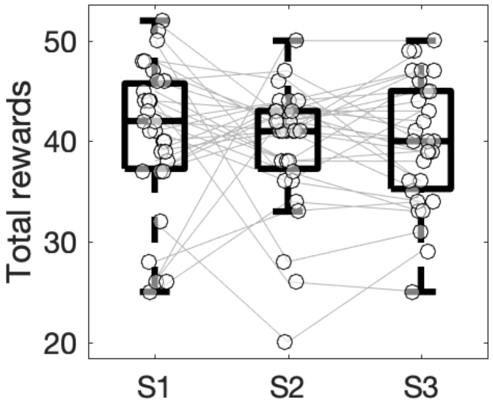

Supplemental Figure S1. Total rewards earned per session (n=31). Bayesian repeated measures ANOVA yielded moderate evidence against an effect of session (BF01=8.41). Lines represent the median, the box covers the upper and lower quartiles, and the whiskers denote the range of datapoints falling within 1.5 times the interquartile range.

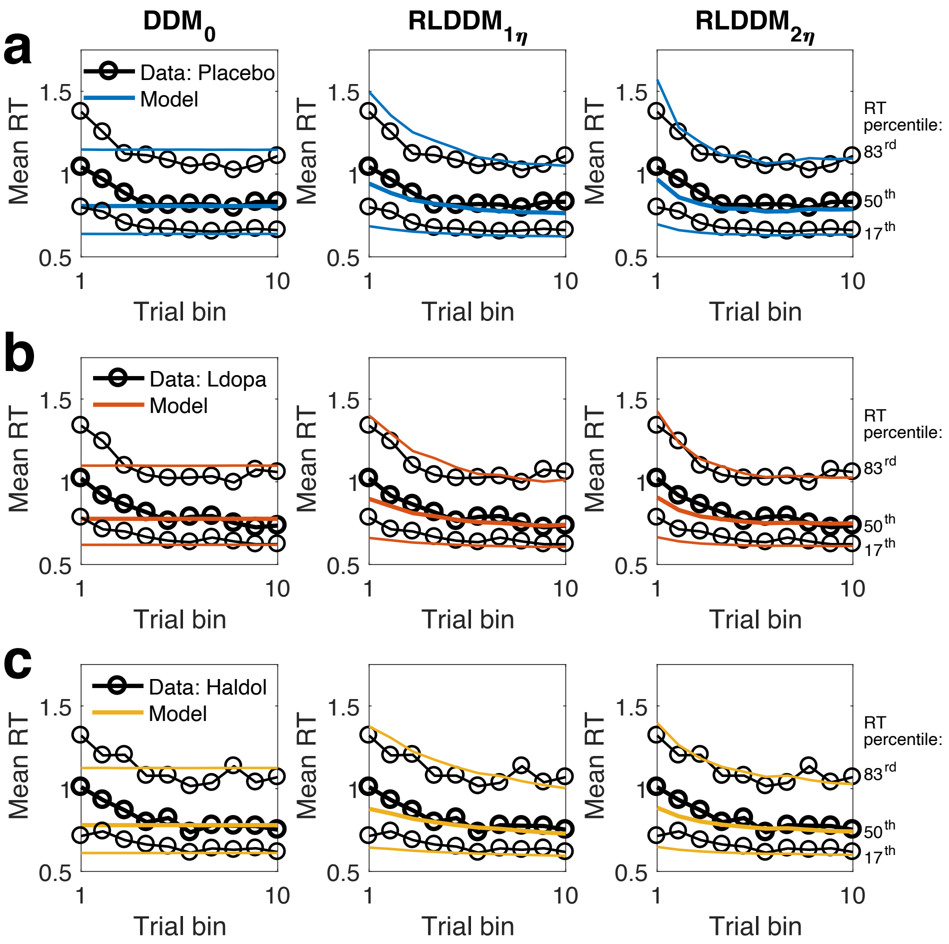

Supplemental Figure S2. Posterior predictive checks for RTs using RLDDM2 (a: Placebo; b: ldopa; c: Haldol). Black lines denote mean RTs across participants per trial bin for median RT (solid black line) and 83rd and 17th percentiles (think black lines). The colored lines show the respective simulations based on the posterior distributions.

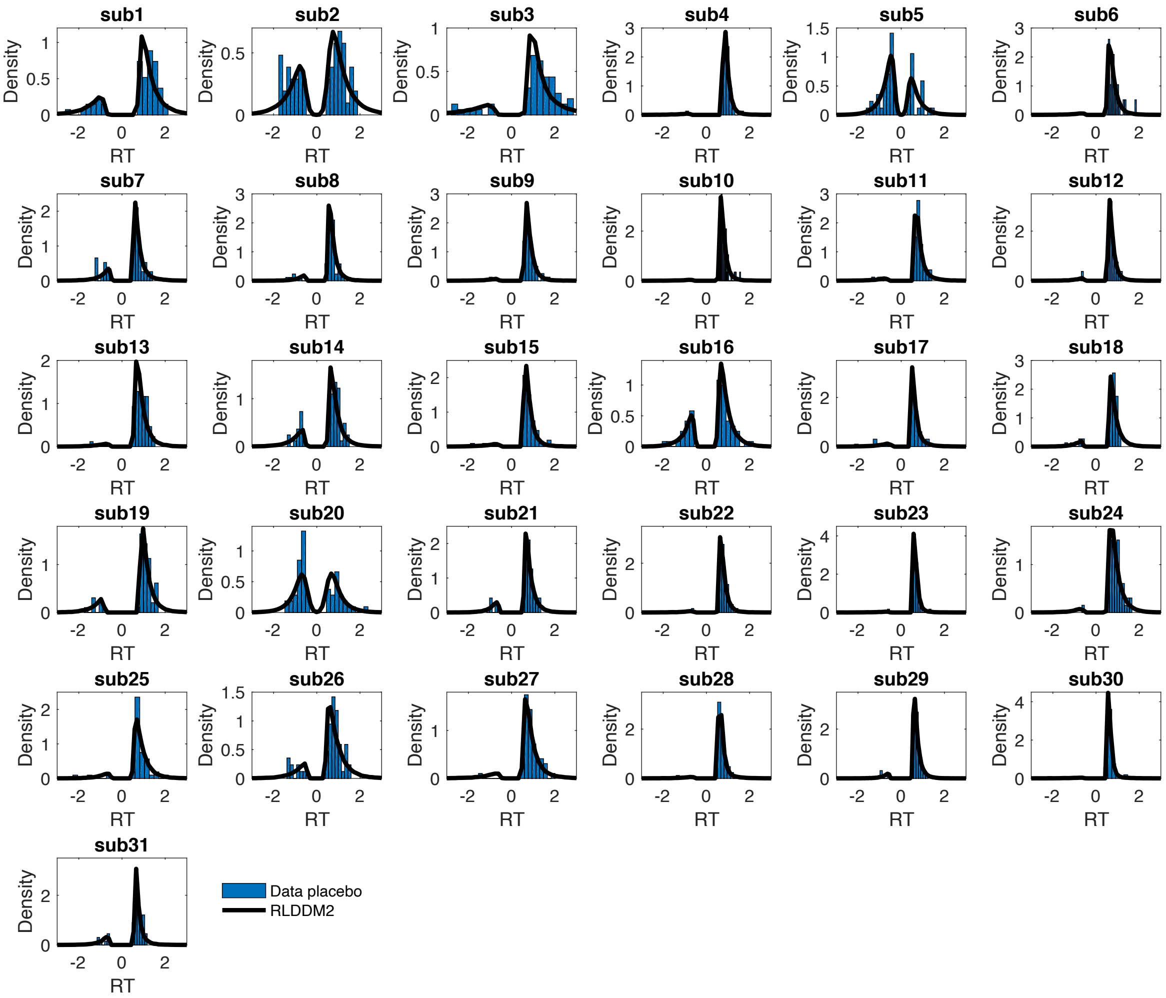

Supplemental Figure S3. Single-participant posterior predictive checks for the placebo condition. Histograms depict single-participant RT distributions (suboptimal choices are plotted as negative RTs). Solid black lines show smoothed histograms across 1k datasets simulated from the RLDDM2 posterior distribution.

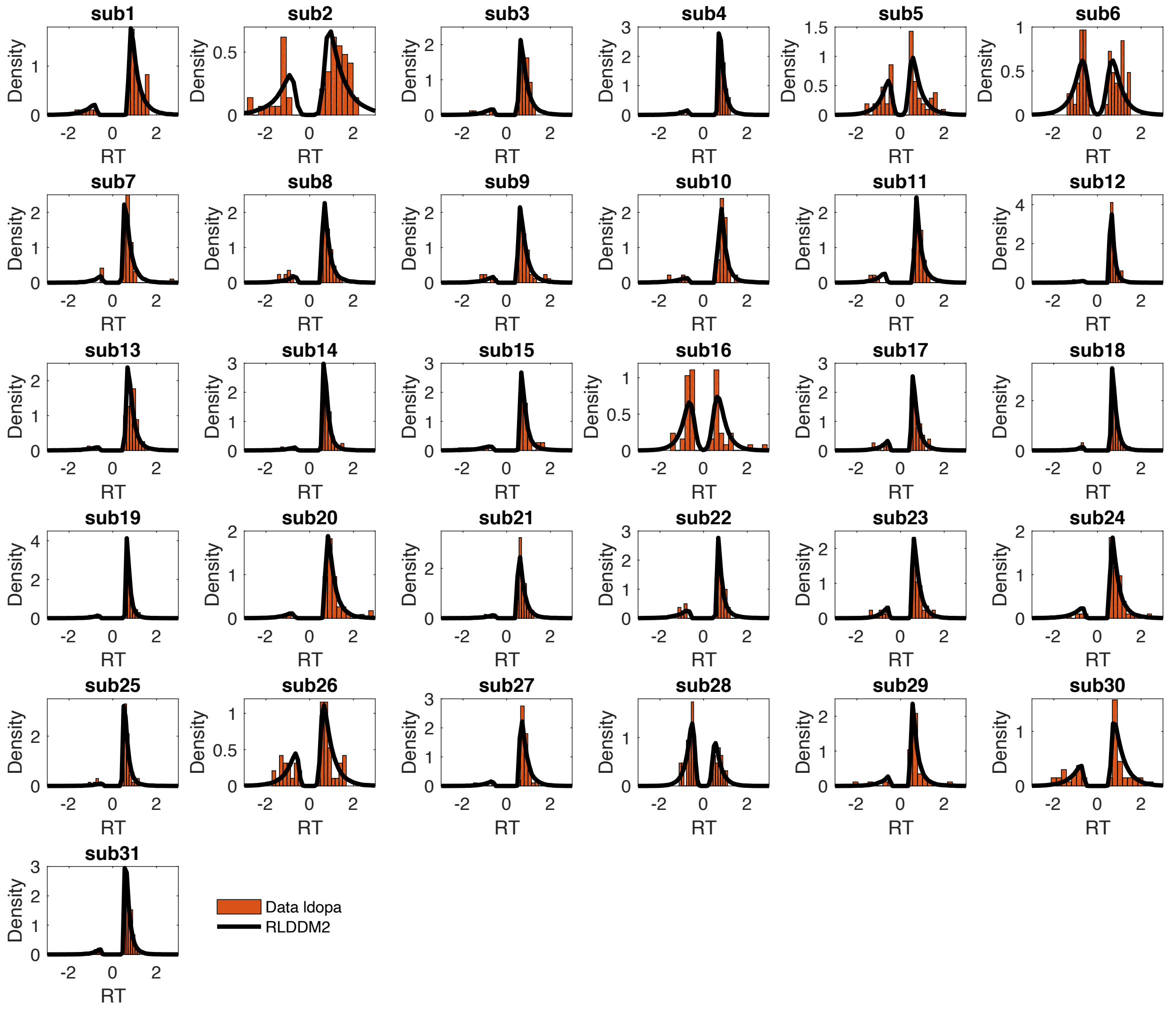

Supplemental Figure S4. Single-participant posterior predictive checks for the ldopa condition. Histograms depict single-participant RT distributions (suboptimal choices are plotted as negative RTs). Solid black lines show smoothed histograms across 1k datasets simulated from the RLDDM2 posterior distribution.

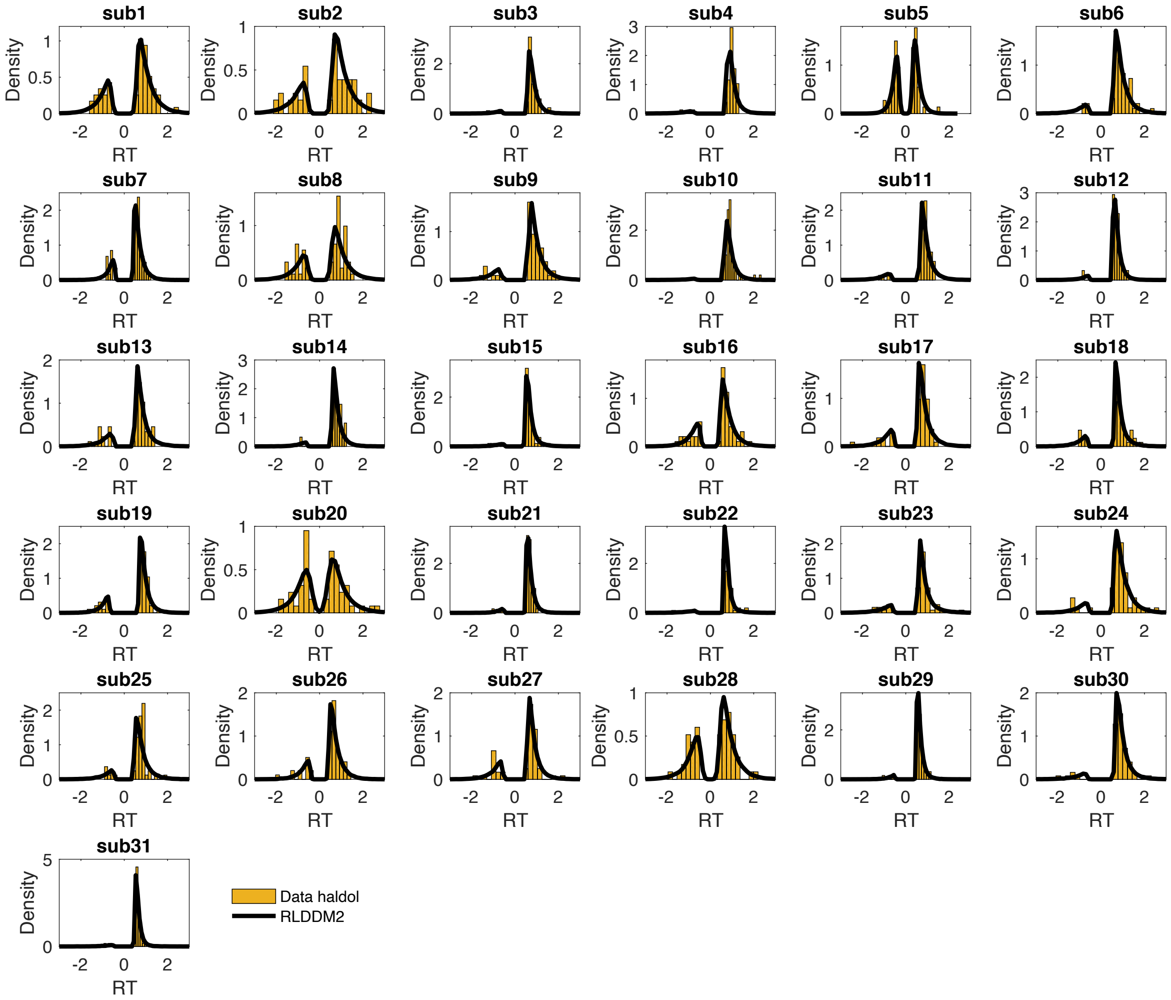

Supplemental Figure S5. Single-participant posterior predictive checks for the haloperidol condition. Histograms depict single-participant RT distributions (suboptimal choices are plotted as negative RTs). Solid black lines show smoothed histograms across 1k datasets simulated from the RLDDM2 posterior distribution.

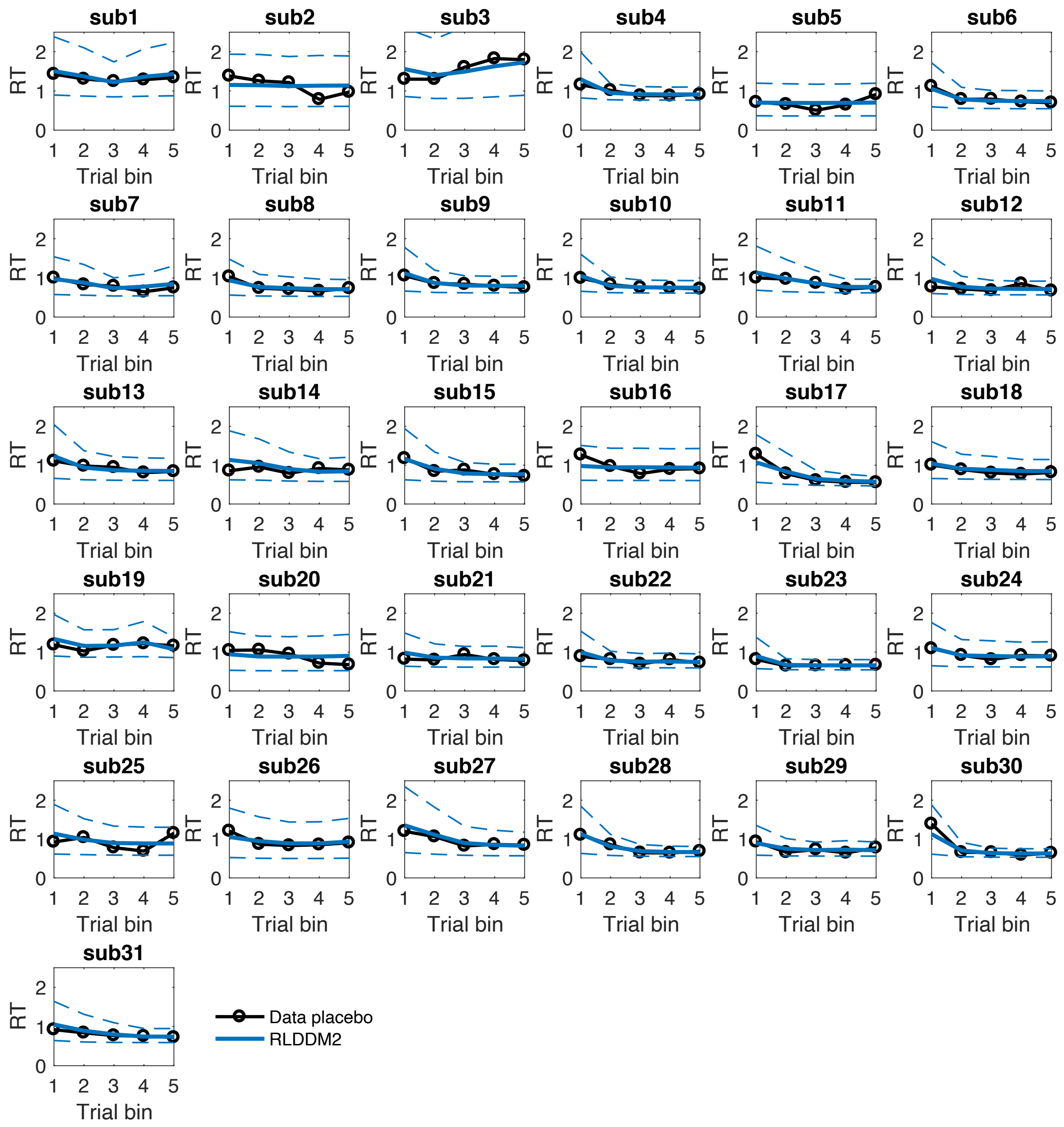

Supplemental Figure S6. Single-participant posterior predictive checks of RLDDM2 for RT changes over the course of learning in the placebo condition. Black lines show mean observed RTs per trial bin. Solid colored lines show mean simulated RTs across 1k posterior samples. Dashed lines show +/- 95% percentiles of the simulations.

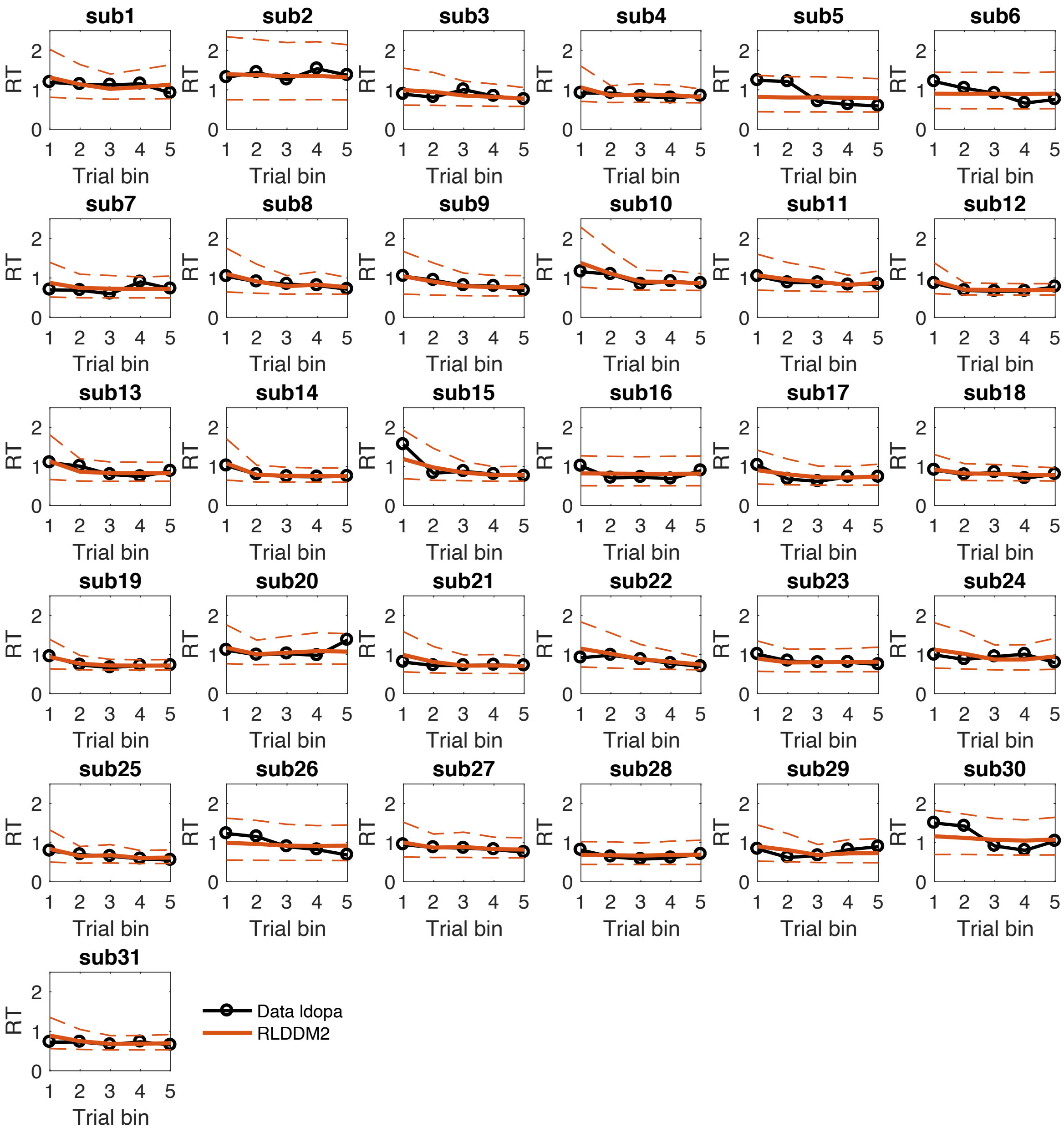

Supplemental Figure S7. Single-participant posterior predictive checks of RLDDM2 for RT changes over the course of learning in the L-Dopa condition. Black lines show mean observed RTs per trial bin. Solid colored lines show mean simulated RTs across 1k posterior samples. Dashed lines show +/- 95% percentiles of the simulations.

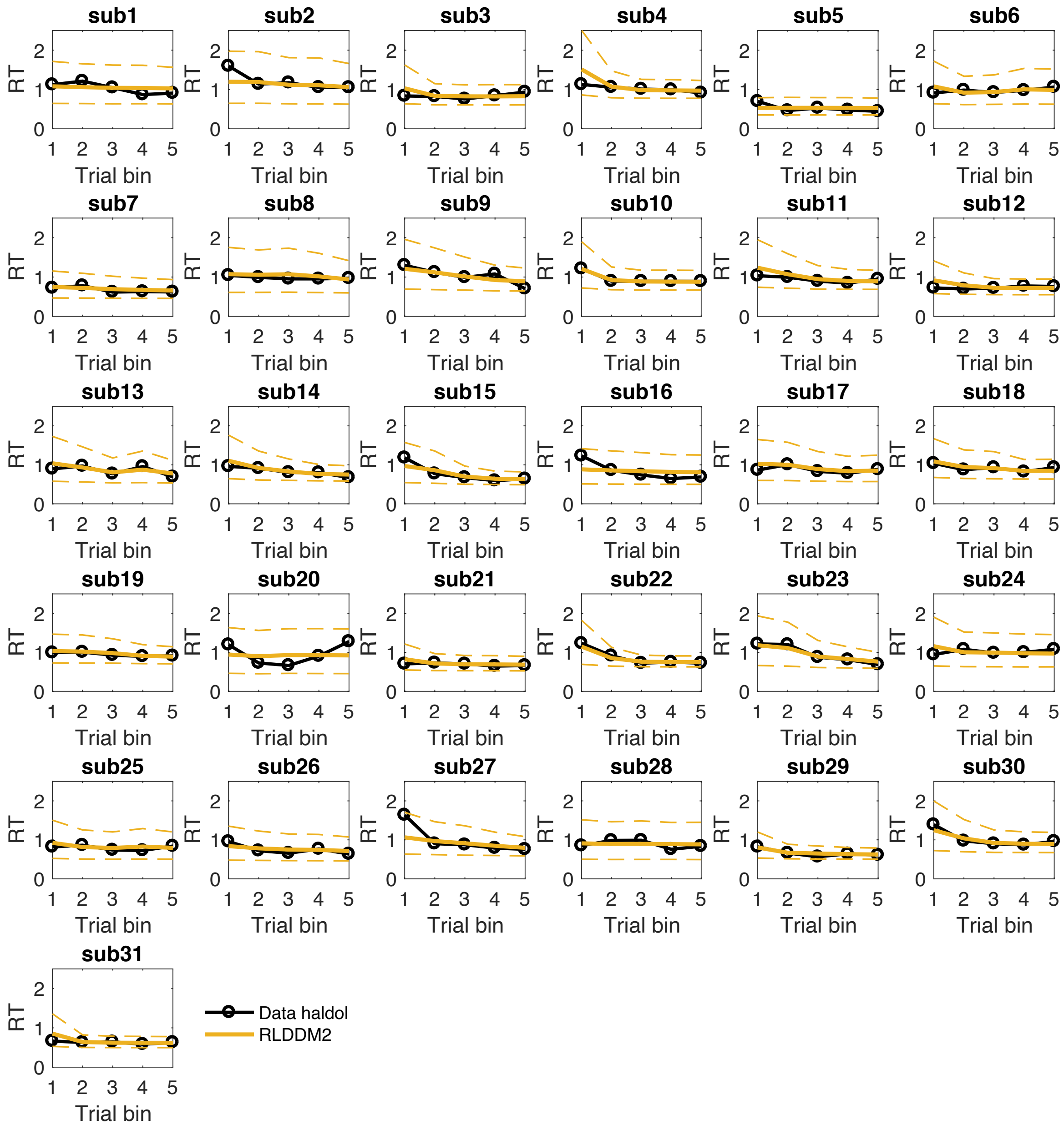

Supplemental Figure S8. Single-participant posterior predictive checks of RLDDM2 for RT changes over the course of learning in the haloperidol condition. Black lines show mean observed RTs per trial bin. Solid colored lines show mean simulated RTs across 1k posterior samples. Dashed lines show +/- 95% percentiles of the simulations.

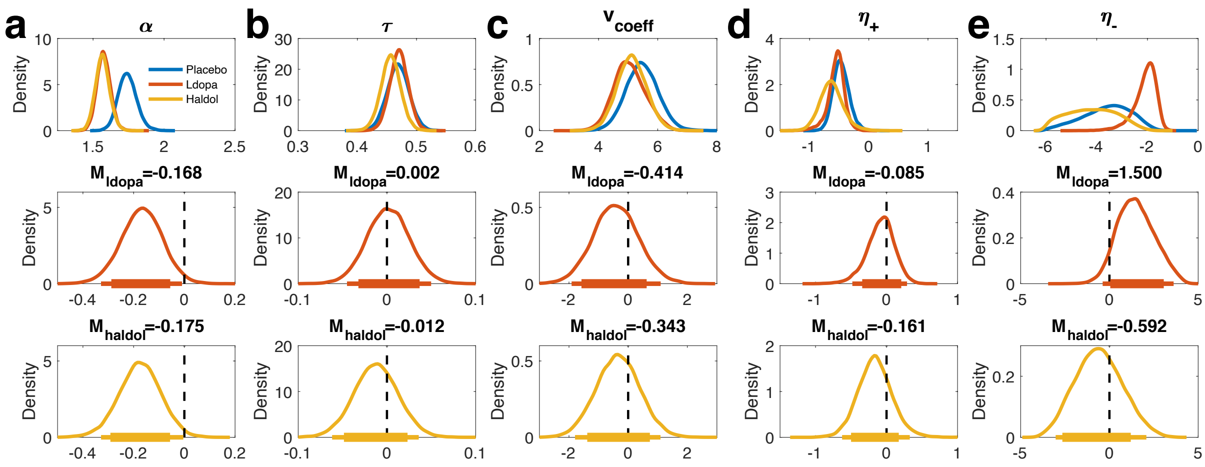

Supplemental Figure S9. Drug effects on RLDDM parameters when modeling each drug condition separately. a: boundary separation (α), b: non-decision time (τ), c: value coefficient of the drift rate (v_{coeff}), d: positive learning rate (η_{+}) in standard normal space, e: negative learning rate (η_{-}) in standard normal space. Top row: Posterior distributions per drug condition. Center row: Posterior distribution differences (Placebo – Ldopa, M_{ldopa} refers to the mean of the difference). Bottom row: Posterior distribution differences (Placebo – Haldol, M_{haldol} refers to the mean of the difference). Solid (thin) horizontal lines denote 85% (95%) highest posterior densities.

Supplemental Table S3. Mean posterior differences in RLDDM2 model parameters (M [95% highest posterior density interval, HDI]) when fitting separate models per drug condition. P(effect<0): posterior probability that the drug effect is < 0. dBF: Bayes factors testing for directional effects (values > 1 quantify the degree of evidence for a reduction in a parameter compared to placebo, compared to the evidence for an increase, and dBF values < 1 reflect the reverse).

RLDDM2 parameter	L-dopa effect			Haloperidol effect		
	M [95% HDI]	*P(effect < 0)*	*dBF (< 0)*	*M [95% HDI]*	*P(effect< 0)*	*dBF (<0)*
α	-.168 [-.329, -.009]	.982	48.544	-.175 [-.329, -.004]	.986	66.667
τ	.002 [-.045, .05]	.477	.959	-.0122 [-.062, .036]	.463	2.161
v_{coeff}	-.414 [-1.912, 1.099]	.708	2.453	-.342 [-1.796, 1.094]	.679	1.954
η+	-.085 [-.482, .289]	.655	1.822	-.161 [-.626, .327]	.764	2.959
η-	1.50 [-.382, 3.617]	.064	.076	.487 [-3.03, 2.081]	.675	2.053

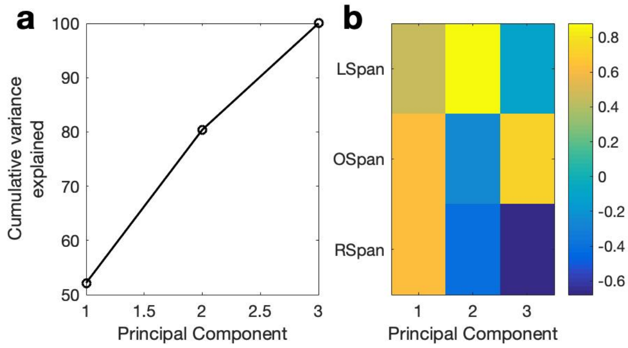

Supplemental Figure S10. Principal component analysis results across working memory measures (listening span, operation span, rotation span). a: Cumulative variance explained across components. The first principal component explained > 50% of variance across measures. b: Principal component coefficients per component and working memory measure. The first component was taken as a measure of working memory capacity.

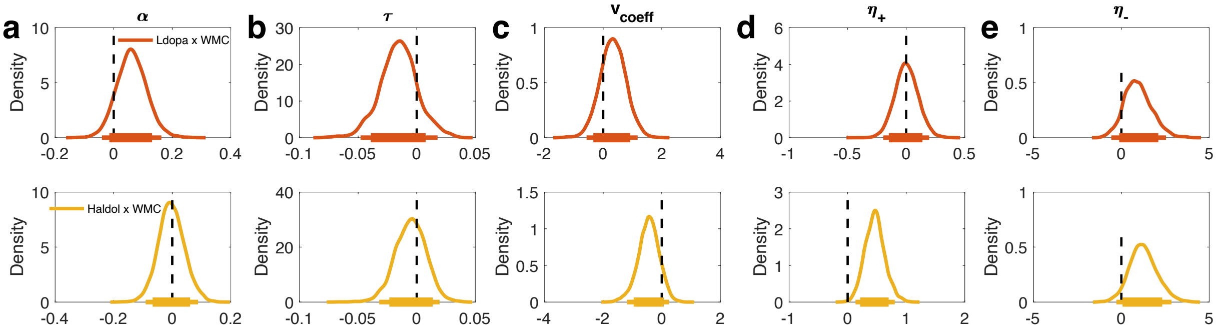

Supplemental Figure S11. Posterior distributions for ldopa-effects (top row) and haloperidol effects (bottom row) modulated by linear effects of working memory capacity (WMC), for each RLDDM parameter.

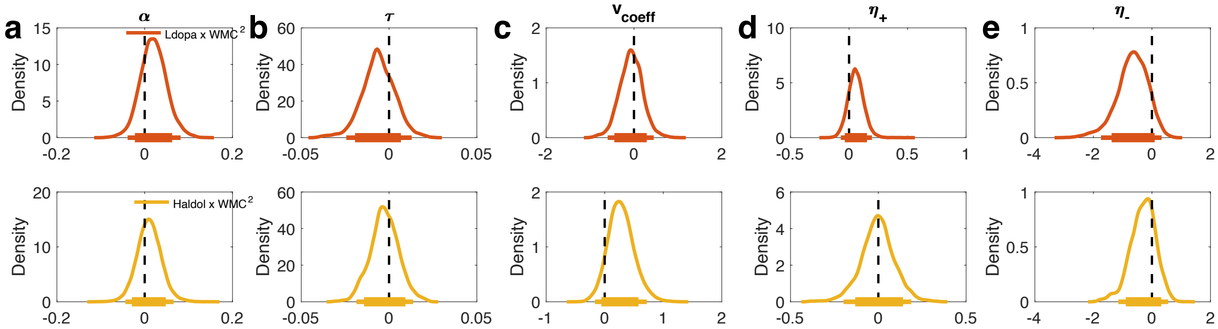

Supplemental Figure S12. Posterior distributions for ldopa-effects (top row) and haloperidol effects (bottom row) modulated by quadratic effects of working memory capacity (WMC), for each RLDDM parameter.

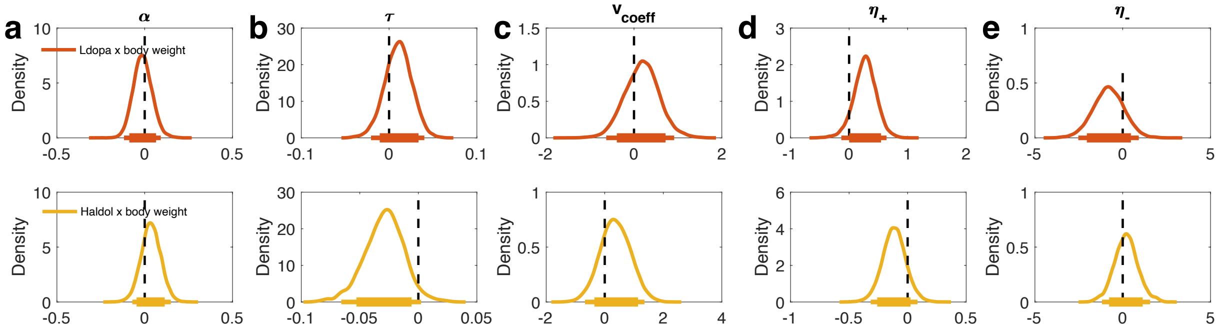

Supplemental Figure S13. Posterior distributions for ldopa-effects (top row) and haloperidol effects (bottom row) modulated by linear effects of body weight, for each RLDDM parameter.

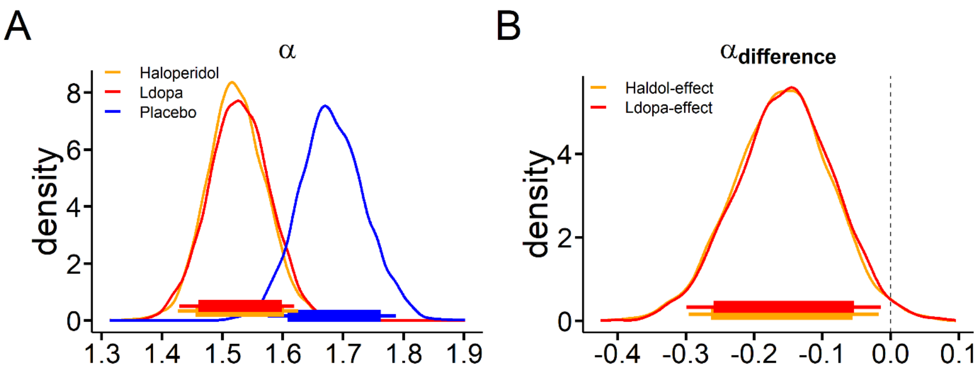

Supplemental Figure S14. Decision threshold results from an RLDDM with constant bounds implemented in the HDDM toolbox reproduced the drug effects on thresholds (α) obtained via JAGS. A: Boundary separation parameter per condition. B: Difference distributions (Haldol – Placebo and Ldopa – Placebo). Solid (thin) horizontal lines denote 85% (95%) highest posterior density intervals.

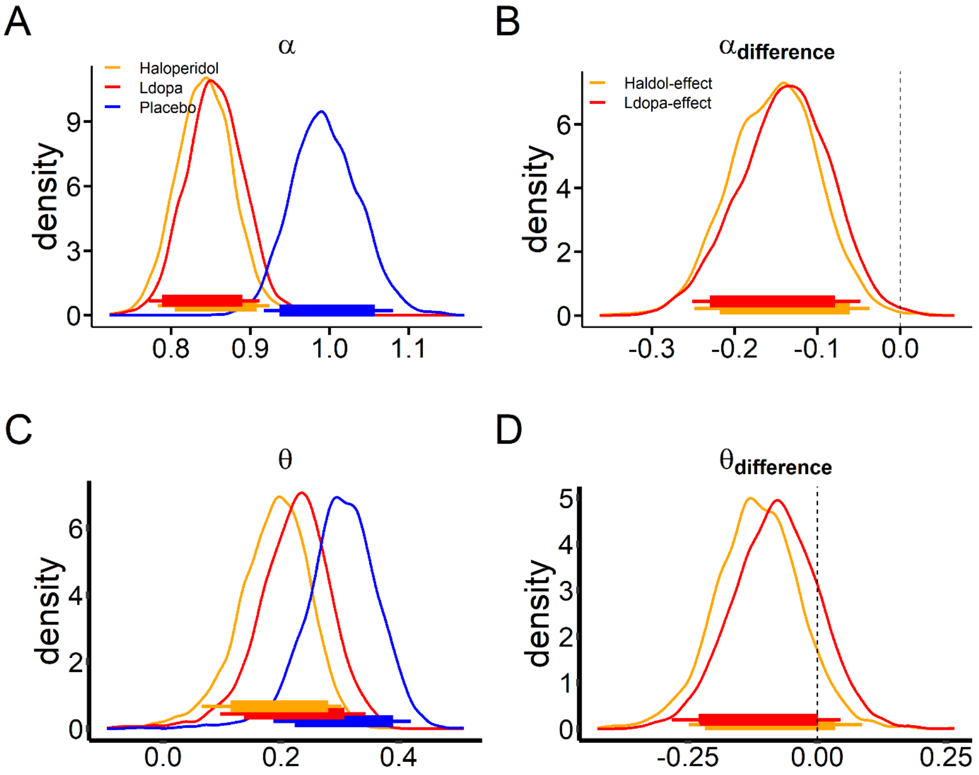

Supplemental Figure S15. Decision threshold results from an RLDDM with linearly collapsing bounds (‘angle’ model) implemented in the HDDM toolbox. A: The overall decision threshold (α) was again reduced under both Ldopa and Haloperidol. B: Difference distributions for α (Haldol – Placebo and Ldopa – Placebo). C: Boundary collapse angle θ per drug. D: Difference distributions for θ (Haldol – Placebo and Ldopa – Placebo). Solid (thin) horizontal lines denote 85% (95%) highest posterior density intervals.

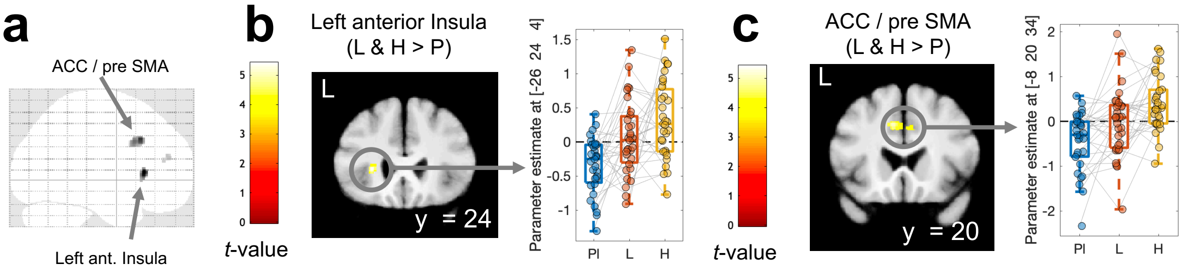

Supplemental Figure S16. FMRI results (n=31). a: Glass brain exploratory whole-brain analysis results (p<.0001, *uncorrected*) testing for higher average value effects under L-dopa & Haloperidol vs. Placebo. This revealed effects in the left anterior insula (b) and the mid cingulate region / pre SMA (c). Boxplots show individual-participant parameter estimates extracted at group-level activation peaks, where lines represent the median, the box covers the upper and lower quartiles, and the whiskers denote the range of datapoints falling within 1.5 times the interquartile range.

Supplemental Table S4. Comparisons of placbo > drug and drug > placebo (exploratory whole-brain analysis, p<.0001, ≥10 voxels). For this exploratory analysis, no correction for multiple comparisons was applied.

Contrast / *Region*	Coordinates	Peak T-value
Average Q-value		
Placebo > Ldopa & Haldol:	No cluster	
Ldopa & Haldol > Placebo:		
Left anterior INS	-26 24 4	5.42
Mid cingulate / pre SMA	-8 20 34	5.04
Left DLPFC	33 46 18	4.14
Chosen-unchosen Q-value		
	No cluster	
Reward prediction error		
	No cluster	

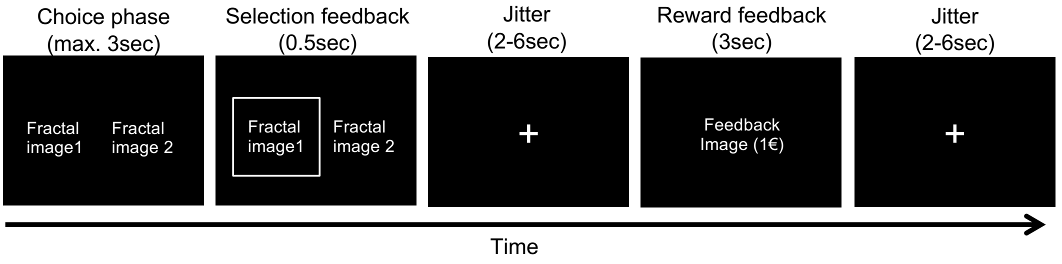

Supplemental Figure S17. Illustration of a single trial from the reinforcement learning task. Stimuli (fractal images, not shown) were presented for a maximum of 3sec, during which participants were free to make their selection. The selection was then highlighted for 500ms, followed by a jitter of variable duration (2-6sec, uniform distribution). Reward feedback (1€ coin image for positive feedback, crossed 1€ coin image for negative feedback) was then presented for 3sec, followed by another jitter of variable duration (2-6sec, uniform distribution). Stimuli consisted of two pairs of abstract fractal images (80%

vs. 20% reinforcement rate), which were presented in randomized order, and participants completed 30 trials per pair.

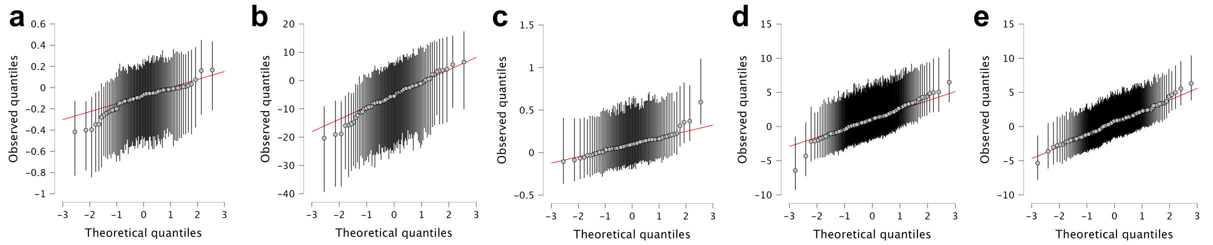

Supplemental Figure S18. Q-Q-plots for all Bayesian repeated-measures ANOVAs with covariates of weight and linear as well as quadratic effects of working memory capacity. a: behavioral accuracy, b: total rewards, c: median RT, d: parameter estimates from left ventral striatum, e: parameter estimates from right ventral striatum.

Supplemental Table S5. Group-mean posterior positive (η_+) and negative (η_-) learning rates used for value and prediction error calculation. These values were taken from RLDDM2 fitted separately to the data from each drug condition.

**Condition**	$\boldsymbol{\eta_+}$	$\boldsymbol{\eta_-}$
Placebo	.320	.0002
L-Dopa	.291	.018
Haloperidol	.265	.00001
