# Supplementary material for: Dopamine regulates decision thresholds in human reinforcement learning in males
Source: Nat Commun. 2023 Sep 4;14:5369. doi: 10.1038/s41467-023-41130-y (PMC10477234; doi:10.1038/s41467-023-41130-y)
Supplement: Supplementary file 3 — Reporting Summary [file 41467_2023_41130_MOESM3_ESM.pdf]

nature portfolio

Corresponding author(s): Jan Peters

Last updated by author(s): Jul 8, 2023

Reporting Summary

Nature Portfolio wishes to improve the reproducibility of the work that we publish. This form provides structure for consistency and transparency in reporting. For further information on Nature Portfolio policies, see our Editorial Policies and the Editorial Policy Checklist.

Statistics

For all statistical analyses, confirm that the following items are present in the figure legend, table legend, main text, or Methods section.

n/a	Confirmed	
☐	☒	The exact sample size (n) for each experimental group/condition, given as a discrete number and unit of measurement
☐	☒	A statement on whether measurements were taken from distinct samples or whether the same sample was measured repeatedly
☐	☒	The statistical test(s) used AND whether they are one- or two-sided<br>*Only common tests should be described solely by name; describe more complex techniques in the Methods section.*
☐	☒	A description of all covariates tested
☐	☒	A description of any assumptions or corrections, such as tests of normality and adjustment for multiple comparisons
☐	☒	A full description of the statistical parameters including central tendency (e.g. means) or other basic estimates (e.g. regression coefficient) AND variation (e.g. standard deviation) or associated estimates of uncertainty (e.g. confidence intervals)
☐	☒	For null hypothesis testing, the test statistic (e.g. F, t, r) with confidence intervals, effect sizes, degrees of freedom and P value noted<br>*Give P values as exact values whenever suitable.*
☐	☒	For Bayesian analysis, information on the choice of priors and Markov chain Monte Carlo settings
☐	☒	For hierarchical and complex designs, identification of the appropriate level for tests and full reporting of outcomes
☐	☒	Estimates of effect sizes (e.g. Cohen's d, Pearson's r), indicating how they were calculated

Our web collection on statistics for biologists contains articles on many of the points above.

Software and code

Policy information about availability of computer code

Data collection	The task was programmed in the Psychophysics Toolbox (version 3.0.12) running in Matlab (2014b).
Data analysis	Imaging data were analyzed using SPM12. Behavioral data were analyzed using JAGS (Plummer, 2003) with the matjags interface, and Matlab 2016, as well as the JASP software package (version 0.16.3). All custom JAGS model code ist available at https://osf.io/8vzgh/. Additional modeling used the HDDM toolbox (version 0.9.8) implemented in Python.

For manuscripts utilizing custom algorithms or software that are central to the research but not yet described in published literature, software must be made available to editors and reviewers. We strongly encourage code deposition in a community repository (e.g. GitHub). See the Nature Portfolio guidelines for submitting code & software for further information.

Data

Policy information about availability of data

All manuscripts must include a data availability statement. This statement should provide the following information, where applicable:

- Accession codes, unique identifiers, or web links for publicly available datasets
- A description of any restrictions on data availability
- For clinical datasets or third party data, please ensure that the statement adheres to our policy

Behavioral data generated in this study as well as fitted models have been deposited on OSF (https://osf.io/8vzgh/). Unprocessed fMRI data are protected and are not available due to data privacy laws. The processed 2nd-level fMRI data are deposited on OSF (https://osf.io/8vzgh/).

Research involving human participants, their data, or biological material

Policy information about studies with human participants or human data. See also policy information about sex, gender (identity/presentation), and sexual orientation and race, ethnicity and racism.

Reporting on sex and gender	We only included participants self-identifying as male. This is noted in the abstract, and discussed in the limitations section of the paper.
Reporting on race, ethnicity, or other socially relevant groupings	n/a
Population characteristics	see below
Recruitment	Participants were recruited via online ads.
Ethics oversight	Study procedures were approved by the local ethics board (Hamburg Board of Physicians, study code PV4720)

Note that full information on the approval of the study protocol must also be provided in the manuscript.

Field-specific reporting

Please select the one below that is the best fit for your research. If you are not sure, read the appropriate sections before making your selection.

☐ Life sciences ☒ Behavioural & social sciences ☐ Ecological, evolutionary & environmental sciences

For a reference copy of the document with all sections, see nature.com/documents/nr-reporting-summary-flat.pdf

Behavioural & social sciences study design

All studies must disclose on these points even when the disclosure is negative.

Study description	We obtained quantitative data from a reinforcement learning task during fMRI, in combination with a pharmacological manipulation.
Research sample	Participants were recruited via online ads and mainly included university students (age 19-35, M=26.85, SD=4.01).
Sampling strategy	Sample size (n=31) was determined based on previous work (e.g. Pessiglione et al., 2006, n=13, Daw et al., 2006, n=14) and we aimed to test at least twice as many participants for replication purposes.
Data collection	Participants performed a computerized reinforcement learning task during fMRI. In addition to the researcher, an MR technician was present. Both the MR technician and the researcher were blind to the drug condition at all times.
Timing	Data was collected in 2014 and 2015.
Data exclusions	Drift diffusion modeling involved the exclusion of the fastest 5% of trials in each participant, as regularly done in this type of work (see e.g. Peters & D'Esposito, Plos Comp Biol, 2020) to ensure that fast outlier trials do not exert an undue influence on the fitted RT distribution, in particular on the non-decision time parameter.
Non-participation	Three additional participants dropped out of the study due to illness or personal reasons, two after the initial baseline session and one after the first fMRI session.
Randomization	It was a double-blind placebo-controlled within-subjects design, with drug order counterbalanced across participants.

Reporting for specific materials, systems and methods

We require information from authors about some types of materials, experimental systems and methods used in many studies. Here, indicate whether each material, system or method listed is relevant to your study. If you are not sure if a list item applies to your research, read the appropriate section before selecting a response.

Materials & experimental systems

n/a	Involved in the study
☒	☐ Antibodies
☒	☐ Eukaryotic cell lines
☒	☐ Palaeontology and archaeology
☒	☐ Animals and other organisms
☒	☐ Clinical data
☒	☐ Dual use research of concern
☒	☐ Plants

Methods

n/a	Involved in the study
☒	☐ ChIP-seq
☒	☐ Flow cytometry
☐	☒ MRI-based neuroimaging

Magnetic resonance imaging

Experimental design

Design type

Event-related design.

Design specifications

During each fMRI session (placebo, l-dopa, haloperidol), participants completed a single run of a total of 60 trials. A trial lasted on average around 14 seconds. choice phase (3 sec), feedback phase (3sec) and successive trials were separated by variable intervals (2-6sec, uniform distribution).

Behavioral performance measures

We recorded choices (which stimulus was selected) and response times.

Acquisition

Imaging type(s)

functional

Field strength

3T

Sequence & imaging parameters

Each volume consisted of 40 slices (2 x 2 x 2mm in-plane resolution and 1-mm gap, repetition time = 2.47s, echo time 26ms). We tilted volumes by 30° from the anterior and posterior commissures connection line to reduce signal drop out in the ventromedial prefrontal cortex and medial orbitofrontal cortex.

Area of acquisition

Whole-brain

Diffusion MRI ☐ Used ☒ Not used

Preprocessing

Preprocessing software

SPM12

Normalization

Normalization was done using the DARTEL toolbox in SPM12.

Normalization template

ICBM152

Noise and artifact removal

Images were realigned and unwarped to account for movement-related effects.

Volume censoring

No volume censoring was performed. Trials which were excluded from computational modeling (see above) or in which participants failed to respond within the response time window (average of .61 (placebo) / .61 (ldopa) / .45 (haloperidol) trials per subject) were modeled as separate error trials.

Statistical modeling & inference

Model type and settings

We set up first-level general linear models (GLMs) for each participant and drug condition. We used GLM1 for all main analyses, and GLM2 to reproduce a key analysis from Pessiglione et al. (2006).

GLM1 included the following regressors:
1) onset of the decision option presentation
2) onset of the decision option presentation modulated by chosen – unchosen value
3) onset of the decision option presentation modulated by (chosen – unchosen value) squared
4) onset of the feedback presentation
5) onset of the feedback presentation modulated by model-based prediction error
6) onset of the decision option presentation for error trials and
7) onset of the feedback presentation for error trials.

To separate out effects of positive vs. negative prediction error coding, as done in Pessiglione et al. (2006), we set up a second first-level GLM.

GLM2 included the following regressors:
1) onset of the decision option presentation
2) onset of the decision option presentation modulated by chosen – unchosen value
3) onset of the decision option presentation modulated by (chosen – unchosen value) squared
4) onset of the feedback for positive prediction errors
5) onset of the feedback for negative prediction errors
6) onset of the decision option presentation for error trials and
7) onset of the feedback presentation for error trials.

GLM3 replaced chosen-unchosen value in GLM2 with the average Q-value across options.

We used a random effects model for the second level analysis (flexible factorial model). For GLM1, this included a single within-subjects factor of drug condition. For GLM2, this included the factors drug condition and prediction error sign.

Effect(s) tested

Figure 5a: Here we tested for a positive effect of average Q-value across drugs (contrast 2 in GLM3, see above).

Figure 5b: Here we tested for a positive effect of chosen - unchosen value across drugs (contrast 2 in GLM1, see above).

Figure 6: Here we tested for a positive effect of reward prediction error across drugs (contrast 5 in GLM1, see above). We then extracted effects from GLM2 (regressors 4 and 5) to examine positive vs. negative prediction error effects in the identified striatal region.

Specify type of analysis: ☐ Whole brain ☒ ROI-based ☐ Both

Anatomical location(s)

We used a single publically available ROI mask (https://www.rnl.caltech.edu/resources/index.html) spanning ventral striatum, vmPFC, PCC and ACC, based on meta-analyses of reward-related effects.

Statistic type for inference

(See Eklund et al. 2016)

voxel-wise

Correction

We used FWE correction across the above-mentioned ROI volume.

Models & analysis

n/a	Involved in the study
☒	☐ Functional and/or effective connectivity
☒	☐ Graph analysis
☒	☐ Multivariate modeling or predictive analysis
